# BAP editorial: Improving the validity and translation of preclinical research

**DOI:** 10.1177/02698811221104064

**Published:** 2022-06-10

**Authors:** Judith A. Pratt, Emma S. J. Robinson, Cathy Fernandes, David Heal, S Clare Stanford

**Affiliations:** 1 Strathclyde Institute of Pharmacy and Biomedical Science (SIPBS), University of Strathclyde, Glasgow, UK; 2 School of Physiology, Pharmacology and Neuroscience, Faculty of Life Sciences, University of Bristol, Bristol, UK; 3 Social, Genetic and Developmental Psychiatry Centre and MRC Centre for Neurodevelopmental Disorders, Institute of Psychiatry, Psychology and Neuroscience, King’s College London, London, UK; 4 DevelRx Limited, Discovery and Development, BioCity, Nottingham, UK; 5 Departement of Neuroscience, Physiology and Pharmacology, University College London, London, UK

The October 2021 edition of *Journal of Psychopharmacology* focussed on the contemporary topic of translational psychopharmacology. Translational research is critical for the development of early interventions and improved therapeutics for mental health conditions. In this respect, the continual updating of information gained from reciprocal forward (preclinical) and reverse (clinical) translational approaches is necessary. Despite the enthusiasm for translational psychopharmacology in the preclinical research community, there are many challenges if we are to achieve the crucial goal of developing therapies with superior efficacy and tolerability over current drugs for neuropsychiatric conditions. At a recent meeting of a preclinical subpanel of the *Journal of Psychopharmacology* editorial board, members debated opportunities to improve the validity and translation of preclinical research models. A particular focus being the importance of understanding the clinical relevance of a model and its readouts and how well the mechanisms associated with those readouts and arising novel drug targets translate to the clinic. Importantly, consideration was given to moving away from pharmacological models towards the development of approaches which recapitulate more clinically relevant readouts. Some key points are summarised below.

## Model validity

A fundamental point in designing a preclinical model for a neuropsychiatric condition, is for preclinical researchers to be fully conversant with existing clinical diagnostic criteria and the symptom domains where therapies are effective or not. Historically, preclinical models have been based upon whether they fall into the category of construct, face or predictive validity. Models based upon the causes of conditions (construct) are the most challenging to develop, given that the causes of many disorders are multifactorial and often not yet fully established. Hence it is unlikely that a disorder can be achieved in a single experimental manipulation but researchers still argue they can recapitulate a complex disorder in a simple model system. For example, there are numerous examples of a single gene manipulation where the authors claim to have reproduced the triad of impairments seen in autism. More promising approaches are now exploring the effects of combinations of risk factors such as genetic and environmental risk factors to enable models of higher construct validity to be developed. In this respect models of higher construct validity can display translational validity.

Models with face validity and predictive validity have been the most widely used for drug discovery. An example of face validity is sensorimotor gating as measured by prepulse inhibition (PPI) where a similar phenomenon can be observed in rodents and humans. Indeed, pharmacological models that produce PPI deficits in rodents are reversed by antipsychotic drugs, which are clinically effective against the positive symptoms of schizophrenia. However, the evidence that antipsychotic drugs reduce PPI alterations in schizophrenia is equivocal, suggesting that these pharmacological models where PPI is evaluated may show predictive validity (in this case to treat the positive symptoms) rather than face validity where a reversal of disrupted PPI in the clinic would be anticipated. It is notable that PPI is not confined to schizophrenia and so may represent a symptom domain relevant to a range of psychiatric conditions and hence may represent face validity for studying this domain.

Historically, there has been a disproportionate prevalence of behavioural tests being described as ‘depression-like’, or ‘schizophrenia-like’, etc. without due consideration of this meaning. The inappropriate application of predictive screens often based upon pharmacological models and efficacy of current treatments as models of a neuropsychiatric disorder (and subsequently for further investigation of the underlying dysfunctional neurobiology) undermines their translational capacity.

With increased knowledge of the neurobiology of neuropsychiatric conditions, there are now opportunities to improve preclinical models for translational research.

Most importantly, consideration should be given to the relevance of the model for the research question being investigated. For example, if it is to understand the neurobiology of a condition, then predictive validity may not be a primary aim. Instead, the extent to which the behaviour and the underlying neural circuitry is analogous to the human condition/particular behavioural domain may be the prime consideration: that is translational validity. If the aim is not only to identify new drugs (predictive validity), but also their effects on specific aspects of the neurobiology of the condition then face and construct validity have to be coupled with translational validity.

In conditions where drugs have been shown to be effective in the clinic, an important test of the predictive nature of a preclinical model (e.g. gene-environment model) and readout (e.g. clinically relevant behavioural domain) is the ability of a drug to ameliorate the altered readout observed in the model. Notably, translational research where information is feed forward and back between clinical and preclinical researchers offers enhanced opportunities for developing new therapies.

## Model readouts

In psychopharmacology research a wide range of behavioural tasks are executed with varying degrees of alignment to human behaviour. More recently, the application of neurophysiological and neuroimaging signatures (broadly defined as intermediate phenotypes), which relate to known aetiology or neurobiology and symptoms, potentially offer improved translation.

The appropriate execution of validated behavioural tasks, rigour in experimental design and statistical analysis and data interpretation are key to better translation.

These include:

*Methodological adjustments*. Modifications to established experimental procedures should be scientifically validated in order to ensure between lab reproducibility as well as avoiding changing the neurobiological context. An example of such a modification being to change a procedure from chronic mild stressors to repeated moderate stressors without explanation or scientific justification. Moreover, such modifications may impact on the ethical guidelines for animal use (See *Journal of Psychopharmacology* Instructions to Authors, Section 2.8).

*Statistical analysis*. It is important for researchers to acquire skills in current practices in statistical analysis which are key to data interpretation. Notably, considerable efforts are being made in improving exploratory data analysis as well as the application of Bayesian models.

*Interpretation of the data*. Behavioural changes should be interpreted in terms of what is observed whether that be for example disruption of sleep, drug self-administration, motor deficit induced by stressor, rather than an anthropomorphised concept such as inferences about the emotional status of the animal, which cannot be substantiated.

*Confounding factors.* Consideration should be given to a range of behaviours being affected by a stimulus (e.g. stressor or drug) and the impact that these may have on a behavioural readout. For example, if locomotor activity is affected by a stimulus could this be a factor in contributing to the behaviour (e.g. motivation in progressive ratio task) being investigated? Another example is behaviour in the ‘open field’ where ‘emotionality’ and locomotor activity interact and should not be treated as independent variables.

### Innovation

The editorial board subpanel noted that there are opportunities for innovations in the development of translational readouts. Clinical and preclinical collaborations can help address the challenges of aligning human subjective experiences with animal behaviour.

In summary, the subpanel concluded with optimism that translational psychopharmacology research will continue to develop and be impactful. Importantly, a unique aspect of the British Association for Psychopharmacology (BAP) is the interaction between clinical and preclinical researchers, offering a particularly strong strategy to translate scientific advances into therapies. Researchers are encouraged to submit quality manuscripts to *Journal of Psychopharmacology* to sustain and promote the reputation of the Journal as being at the forefront of translational research.

